# Soft X-ray spectromicroscopic proof of a reversible oxidation/reduction of microbial biofilm structures using a novel microfluidic in situ electrochemical device

**DOI:** 10.1038/s41598-024-74768-9

**Published:** 2024-10-14

**Authors:** Pablo Ingino, Haytham Eraky, Chunyang Zhang, Adam P. Hitchcock, Martin Obst

**Affiliations:** 1 Experimental Biogeochemistry, BayCEER, University of Bayreuth, 95448 Bayreuth, Germany; 2 Chemistry & Chemical Biology, McMaster University, Hamilton, ON Canada; 3 Chemical Engineering, McMaster University, Hamilton, ON Canada

**Keywords:** In situ liquid phase electrochemistry, Soft X-ray spectromicroscopy, STXM, Electrochemistry, K_2_Fe(CN)_6_, K_3_Fe(CN)_6_, CO_2_ reduction catalyst, Fe(II)-oxidizing bacteria, Element cycles, Carbon capture and storage, Metal-organic frameworks, Electrocatalysis

## Abstract

In situ electrochemistry on micron and submicron-sized individual particles and thin layers is a valuable, emerging tool for process understanding and optimization in a variety of scientific and technological fields such as material science, process technology, analytical chemistry, and environmental sciences. Electrochemical characterization and manipulation coupled with soft X-ray spectromicroscopy helps identify, quantify, and optimize processes in complex systems such as those with high heterogeneity in the spatial and/or temporal domain. Here we present a novel platform optimized for in situ electrochemistry with variable liquid electrolyte flow in soft X-ray scanning transmission X-ray microscopes (STXM). With four channels for fluid control and a modular design, it is suited for a wealth of experimental conditions. We demonstrate its capabilities by proving the reversible oxidation and reduction of individual microbial biofilm structures formed by microaerophilic Fe(II)-oxidizing bacteria, also known as twisted stalks. We show spectromicroscopically the heterogeneity of the redox activity on the submicron scale. Examples are also provided of electrochemical modification of liquid electrolyte species (Fe(II) and Fe(III) cyanides), and in situ studies of electrodeposited copper nanoparticles as CO_2_ reduction electrocatalysts under reaction conditions.

**Supplementary Information:**

The online version contains supplementary material available at 10.1038/s41598-024-74768-9.

## Introduction

Oxidation and reduction reactions as well as electron transfer between (micro-)organisms, organic matter or abiotic phases such as minerals are fundamental processes in the environment and technical systems. They are of key interest in various fields of sciences including environmental sciences, catalysis, material science and battery development. Despite fast-increasing interest, the study of such processes often poses major technical challenges, in particular when considering their preferred occurrence on submicron- and nanoparticles, or in thin surface layers of such particles.

In this study, we focus on microbial Fe(II)-oxidation, which is an essential part of environmental Fe-cycling^1^. In Fe(II)-rich circum-neutral low oxygen environments, microaerophilic Fe(II)-oxidizing bacteria are capable of competing with abiotic Fe(II)-oxidation. Such bacteria often form sophisticated biopolymer structures such as twisted stalks or sheaths that first complex iron ions and then act as a template for the nucleation and subsequent precipitation^2^ or recrystallization of the resulting Fe-oxyhydroxides^3^. Whereas first models for the cell-internal electron flow have been developed^4,5^, the cell-external mechanisms of Fe(II) oxidation are still not fully understood. Recently, a strong influence of organic ligands on biotic and abiotic Fe(II)-oxidation has been demonstrated^6^. Thus, the mechanism of encrustation of twisted stalks might involve:

(i) electron conductance by the organic polymer of the twisted stalk.
(ii) electron transfer by redox reactions of functional groups on the organic polymer of the twisted stalks, such as quinones or phenolic groups with Fe.
(iii) electron transfer and redox-activity of Fe-ions that are complexed by organic functional groups of the twisted stalk.

On the macroscopic scale, electrochemical approaches can be used to study the underlying mechanisms of these processes. By cyclic voltammetry (CV) and related approaches, the threshold potentials of the oxidation and reduction reactions of the individual components can be identified to further our understanding of electron flow under the macroscopic environmental conditions where these bacteria grow. Unfortunately, microaerophilic Fe(II)-oxidizing bacteria grow in heterogeneous environmental biofilms. Thus, bulk electrochemical measurements give ambiguous results which cannot easily be interpreted for the identification and quantification of electron transfer mechanisms. Therefore, electrochemical sample characterization or manipulation, combined with analytical microscopy at the sub-µm or nm scale, is needed. Such tools should allow for imaging and potential-dependent identification and quantification of the involved redox-active species. Synchrotron-based scanning transmission (soft) X-ray microscopy (STXM) provides selective imaging of chemical species and spectroscopy with tens of nm spatial resolution^7^. It is compatible with both vacuum and atmospheric pressure and allows for in situ studies in aqueous environments. Thus, if it is combined with liquid flow and electrochemical capabilities, STXM is ideally suited for studies of microbial Fe(II)-oxidation. In fact, in situ electrochemical-STXM is an emerging tool for studies of redox-active materials in material science^8^, battery^9^ and catalyst research^10^, and also in environmental research^11^, where the term “in situ” refers to a sample environment similar to the conditions wherein the electrochemical changes occur in the system under study.

Both environmental and materials science studies have in common that the requirements for in situ or *operando* spatially resolved analytics are often at the technical limit: In situ electrochemical cells typically have extremely small volumes (typically < 2000 μm^3^). The combined electrolyte and solid sample thickness must be of the order of a few µm to maintain soft X-ray transparency. Thus, for electrochemical experiments/processes that are consuming dissolved redox-active species, it is necessary to maintain a constant electrolyte flow through the cell to keep the reaction ongoing. This is particularly important for studies at (environmentally relevant) low concentrations of educts, electrolytes, or pH buffers. It may also be necessary to remove unwanted detrimental products. Although in situ electrochemical STXM was pioneered in the 1990’s, the earlier setups were not designed for establishing constant flow conditions^8,10^, or for operation over prolonged periods of time.

Other recently reported in situ electrochemical STXM setups designed for flow-through operation^12^ provided very valuable results and experience with such systems, but technically do not allow for replacement of the electrolyte at efficient timescales (i.e. within minutes). Such electrolyte changes can be required either for experimental reasons (e.g. to stop reactions at certain conditions, or to stabilize a product of an electrochemical reaction) or to make an efficient use of precious synchrotron measurement time. The main reason is that, with only one inlet and one outlet channel, the entire volume of electrolyte residing in the connecting tubing needs to be pumped through a microfluidic electrochemical cell of a few µm thickness.

Our goal is to study individual, extra-cellular components of electrochemically active biofilms, namely twisted stalks that are composed of organic matter, complexed Fe-ions and associated Fe-oxyhydroxide mineral phases. Such structures are heterogeneous down to the tens of nm scale^13^. The identification, spatial mapping, and quantification of the redox properties of such complex materials require the chemical sensitivity, quantitation, and mapping capabilities of soft X-ray STXM that allow for analyzing thin, small samples at high spatial resolution. Additional specific requirements are the aforementioned described features: (1) continuous, controllable flow, (2) in situ electrolyte exchange to e.g. switch between direct and mediated electrochemical reduction^14^, and (3) easy device adaptability (e.g. to different beamlines, or changing experimental requirements).

Here we report implementation of a novel design of an in situ flow electrochemistry platform for soft X-ray STXM, based on a philosophy of modularity, flexibility, and expandability. We demonstrate the performance of this in situ electrochemistry platform under a variety of experimental conditions including liquid phase electrochemical reactions in aqueous solution, electrodeposition from solution, in situ characterization of electrocatalysts and in situ chemical and redox speciation of individual, heterogeneous composite samples:

We first use potassium ferricyanide and potassium ferrocyanide exemplarily to demonstrate the suitability of the platform to study solution electrochemical reactions and the ability of rapid electrolyte change. These compounds have been used as a redox shuttle system^15^ in various applications such as thermogalvanic cells^16^, photoanodes^17^, batteries^18^, and dye sensitized solar cells^19^.

In a second step, we use the device to study in situ electrodeposition of copper nanoparticles on a Au electrode, followed by measurement of changes in the particles as they act as electrocatalysts for the CO_2_ reduction (CO_2_R) reaction. In this context, the device has been used for in situ analytical electrochemical spectromicroscopy using both STXM^20^ and spectro-ptychography^21^.

Finally, we demonstrate the capabilities and scientific merits of this STXM-electrochemistry platform by a detailed study of the chemical and redox speciation of complex and heterogeneous samples that are deposited individually on an electrode with a micromanipulator. Here we study twisted stalks that are composite structures of organic polymers and iron minerals formed by Fe(II)-oxidizing bacteria in environmental biofilms.

## Results

### Concept, design & fabrication of the modular in situ flow electrochemistry device

The design principles and development process of the device were based on the goal of creating a modular, expandable in situ electrochemistry platform with a high degree of flexibility to adapt to different experimental needs. The design shares some aspects with an earlier effort^12^ (e.g. the electrode chips) but it is based on a microfluidic rather than a 3D printing approach.

**Fig. 1:**
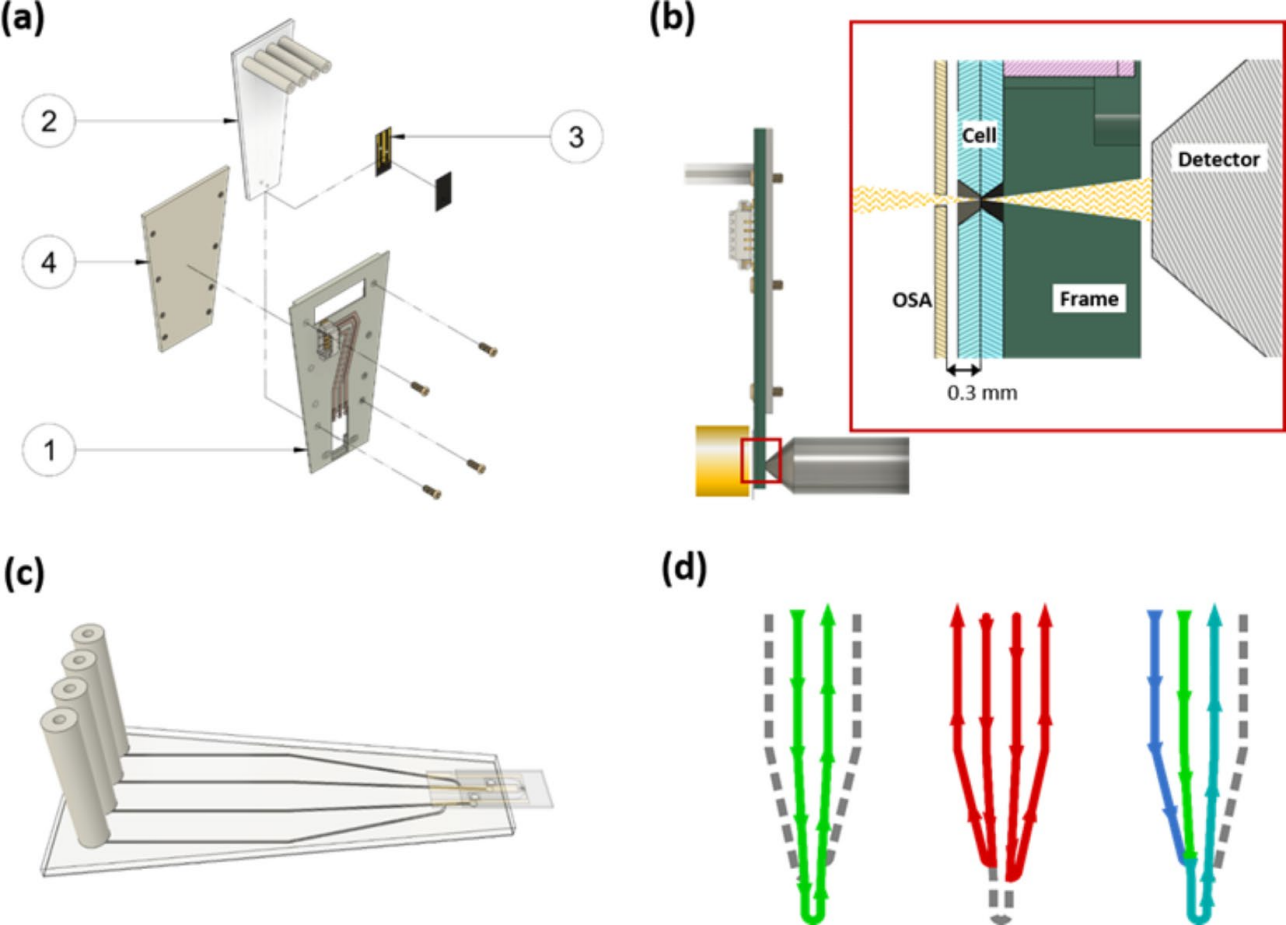
Modular in situ flow electrochemistry platform. (**a**) Individual components and assembly. Mounting frame (1) with electrical circuitry; fluidic chip (2); electrochemical flow cell (3); retaining plate (4). (**b**) Representation of the platform mounted inside a STXM (ambient STXM at CLS) illustrating the special constraints. Drawn to scale with sample – order sorting aperture (OSA) separation of 300 μm, a typical value at the C 1s edge. (**c**) Fluidic chip and electrochemical cell are covalently bound and represent a sealed unit for electrolyte flow and control. (**d**) examples of fluid control possible in the present channel design. From left to right, normal flow conditions through the cell; bypassing the cell for improved speed when changing the electrolyte; spiking an electrolyte; or mixing two electrolytes by using both input channels simultaneously.

Figure 1 presents an overview of the in situ electrochemistry platform. The assembly consists of four main components. (1) the mounting frame, (2) the fluidic chip, (3) the electrochemical flow cell, and (4) the retaining plate (Fig. 1a). The mounting frame is a circuit board designed to slide into the 3-pin kinematic mount used in many soft X-ray STXMs^22^. Integrated into this frame is the circuitry for electrical connectivity. The electrochemical flow cell^12^ and the fluidic chip are covalently bound together using plasma bonding and function as one module (Fig. 1c) that fits in the mounting frame. It is held in place by the retaining plate. A visual representation of how the different modules fit together is given in Fig. 1a. Supplementary Fig. 1 provides dimensions and sketches of the 3 different uses described in this paper. Supplementary Movie 1 provides a 3D representation of all parts and their assembly. Figure 1b displays the schematic of the device mounted inside a STXM, showing the spatial constraints between sample and order sorting aperture (OSA) which must be ~ 300 μm in order to perform STXM at the C 1s edge.

With two input and two output channels in the fluidic chip design used in this study, a variety of different flow conditions can be achieved (Fig. 1d). The flow can be directed through the cell, but the cell can also be bypassed, e.g. to facilitate fast electrolyte exchange. More complex flow scenarios are possible, e.g. where a solution is spiked with an additional analyte/reactant through the second inlet port, or the flow is alternated between two different electrolytes.

The cross-sections of the microfluidic channels are 200 × 100 μm, which is a compromise between minimizing the total volume and accelerating the exchange of electrolyte.

**Fig. 2:**
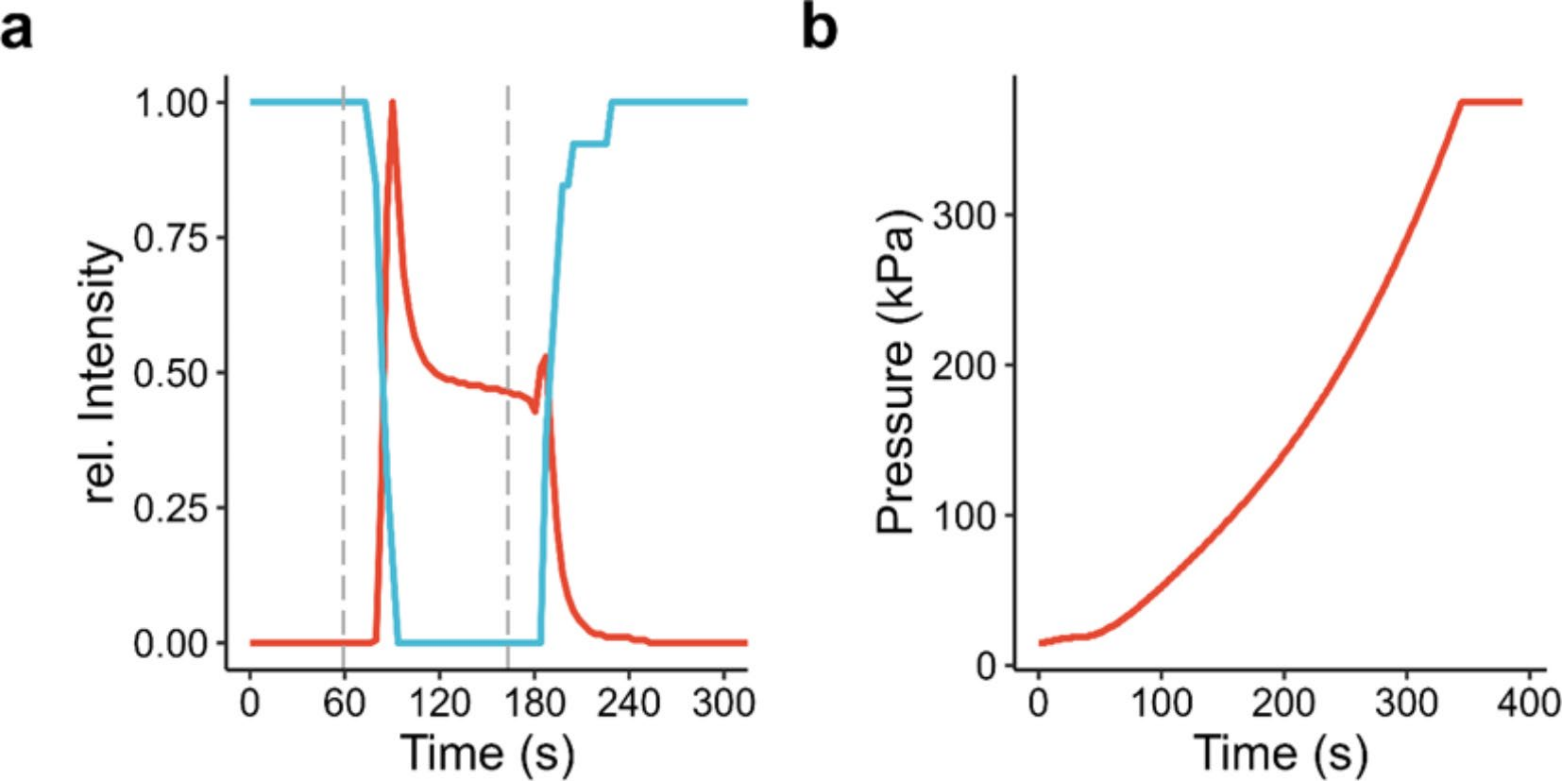
Properties of the modular in situ flow electrochemistry platform. (**a**) Fluid control inside the electrochemical cell. Shown are fluorescence intensity profiles of aqueous solutions of resorufin (blue) and calcein (red) recorded by LSM in the electrochemical cell. Dashed vertical lines indicate when the flow was switched from the resorufin solution to the calcein solution and vice versa. (**b**) Pressure profile showing pressure vs. time of the closed system under constant pumping.

These capabilities were demonstrated by measuring the fluorescence signal of two different fluorophores (resorufin, calcein) in the in situ device by Laser Scanning Microscopy (LSM). The flow of the two fluorophore solutions was controlled separately via syringe pumps in a configuration as depicted in Fig. 1d right. As shown in Fig. 2a, the four-channel design allows for fast, controlled, and quantitative exchange of solutions inside the electrochemical flow cell, which had a thickness of 3.3 μm in the window region. The results show the ability to exchange the electrolyte inside a cell while maintaining in situ conditions within approximately 2 min.

The introduction of air bubbles into a microfluidic system during assembly or the formation of gas bubbles during electrochemical experiments are potential sources of problems. In such cases, the ability to bypass the electrochemical cell has proven invaluable to allow for efficient operation of the device during time-limited synchrotron experiments. Gas bubbles occurring during experimentation can be efficiently removed by flushing either the inlet or the outlet channel loop, while the setup is kept in situ (i.e. without the need of disassembling the experimental setup).

Leakage tests were performed in two ways. First, by introducing ink solution into a closed off device, the maximum overpressure before leakage was measured at 375 kPa (Fig. 2b). No leaks were observed within the device itself. Leakage instead occurred at the connection between the fluorinated ethylene propylene (FEP) and silicone tubing, suggesting the device itself can tolerate even greater pressure differences. Second, the device was successfully operated inside a STXM tank at < 18.5 Pa without the occurrence of leaks (Supplementary Fig. 2).

### STXM in situ flow electrochemistry of ferricyanide & ferrocyanide in solution

The capability of the device to perform in situ flow electrochemistry experiments was demonstrated by running cyclic voltammetry on two different solution analytes, ferricyanide and ferrocyanide. Ferricyanide/ferrocyanide redox electrochemistry and STXM spectroscopy measurements were conducted sequentially in the same device on two separate redox-systems by exchanging the ferrocyanide solution with a ferricyanide solution while the system was electrochemically active.

**Fig. 3:**
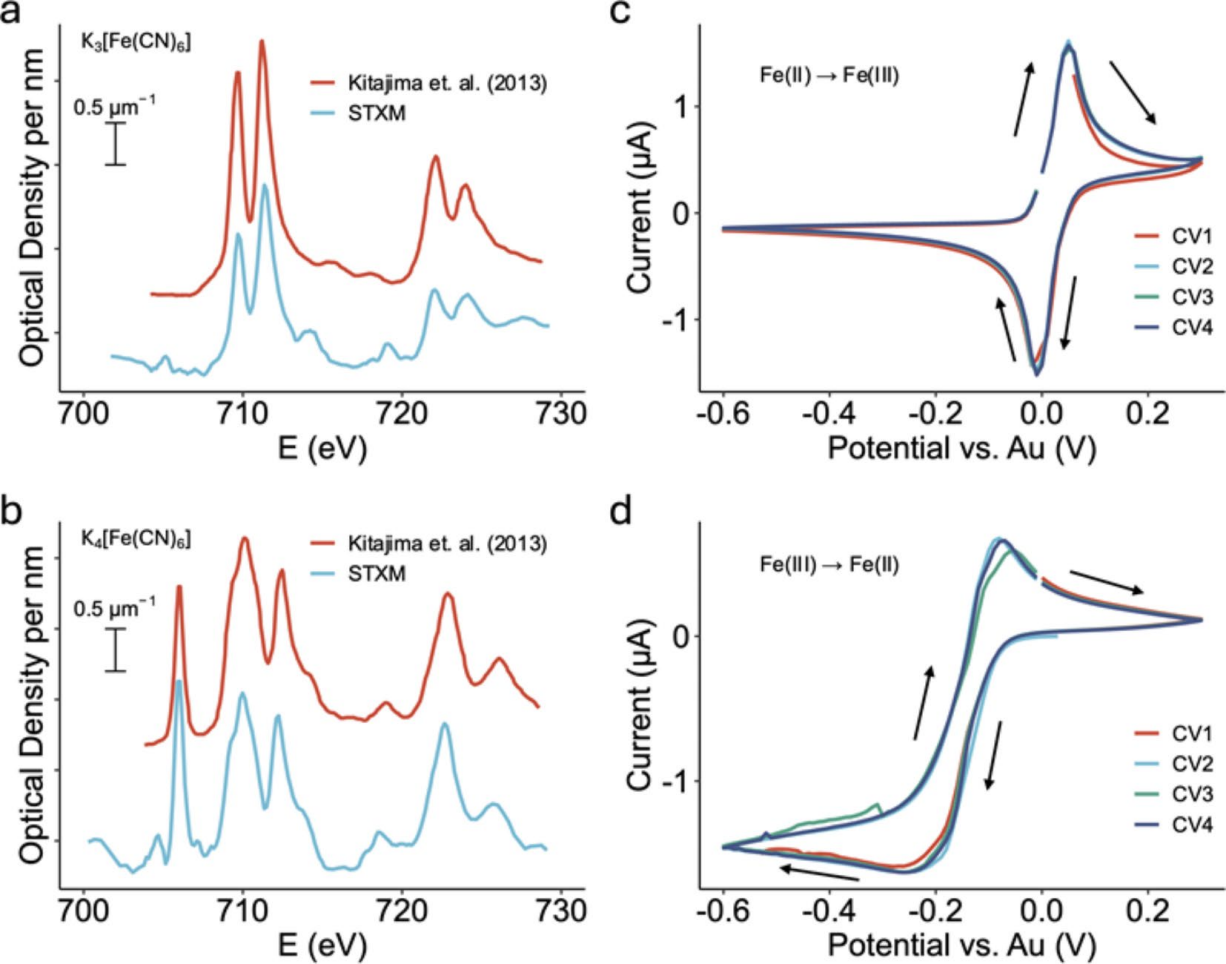
In situ flow electrochemistry STXM of potassium ferricyanide (K_3_[Fe(CN)_6_] (aq)) and potassium ferrocyanide (K_4_[Fe(CN)_6_] (aq)). Fe L_3,2_ X-ray absorption spectrum of (**a**) 0.1 M K_3_[Fe(CN)_6_] solution (no applied potential) and (**b**) 0.1 M K_4_[Fe(CN)_6_] solution (no applied potential) measured in this study, each in comparison to the spectra of the corresponding solid reported by Kitajima et al.^23^ (**c**) In situ cyclic voltammetry of 0.1 M potassium ferrocyanide solution measured with a scan rate of 20 mVs^−1^. (**d**) In situ cyclic voltammetry of 0.1 M potassium ferricyanide solution measured with a scan rate of 20 mVs^−1^.

Figure 3a-b presents the measured Fe L_3,2_ spectra of ferri- and ferrocyanide ions measured in 0.1 M aqueous solution in the in situ device, in comparison to spectra of K_4_Fe(CN)_6_.3H_2_O (s) and K_3_Fe(CN)_6_ (s) digitized from the literature^23^. The energy scale was set by assigning the position of the first peak in the Fe L_3,2_ spectrum of K_3_[Fe(CN)_6_] to 706.2 eV^23^. As summarized in Supplementary Table 1, the energies of the main spectral features match those reported in the literature^15,23,24^. The assignments of these spectra, which have been discussed extensively in the literature^25–27^ are complex since the effects of spin-orbit splitting, crystal field splitting, covalency, and ligand-metal charge transfer (both σ donation and π back donation), as well as core hole relaxation effects, all must be taken into account.

The high quality of the spectra reported in Fig. 3a-b was achieved by using a much higher solution concentration than would typically be used for electrochemical experiments. In fact the spectra of ions in aqueous solution can be measured in the in situ device at much lower concentrations with lower precision but sufficient for chemical identification. For example, Supplementary Fig. 3 presents the Cu L_3_ spectrum of a 0.01 M CuSO_4_ (aq) solution, measured in the in situ device.

Figure 3c-d presents the cyclic voltammetry (CV) results performed in situ in the potential window of + 0.3 to − 0.6 V_Au_ with a scan rate of 20 mVs^− 1^. In the in situ cell the voltage is measured relative to a Au pseudo-reference (V_Au_) which is the potential between the counter electrode (CE) and the reference electrode (RE), both of which are Au. Supplementary Fig. 4 section presents the voltage relationship between the in-cell reference (V_Au_) and Ag/AgCl by comparing beaker-scale CVs measured using the 3-electrode system (with Au as pseudo reference) with that measured using a standard Ag/AgCl reference electrode. The potential difference is then converted to that of the reversible hydrogen electrode (RHE) using the Nernst equation^28^ as discussed in Supplementary Fig. 4 section. The relationship between the Au-pseudo reference and RHE is:

1 $${\text{V}}_{{{\text{RHE}}}} = {\text{V}}_{{{\text{Ag/AgCl}}}} + 0.63 = {\text{V}}_{{{\text{Au}}}} + 0.84$$

In the in situ CV of potassium ferrocyanide (K_4_[Fe(CN)_6_]) (Fig. 3c), the anodic peak (oxidation) occurred at + 0.05 V_Au_ (+ 0.89 V_RHE_) while the cathodic (reduction) peak occurred at − 0.01 V_Au_ (+ 0.83 V_RHE_). For potassium ferricyanide (K_3_[Fe(CN)_6_]) the CV was significantly different (Fig. 3d), with peak displacements and a stronger reduction wave and a weaker oxidative wave compared to the CV of ferrocyanide. The anodic and cathodic peaks of ferricyanide appeared at − 0.07 V_Au_ (+ 0.77 V_RHE_) and − 0.26 V_Au_ (+ 0.58 V_RHE_), respectively. The − 0.12 V shift between the anodic peaks of the two species is much larger than that in CVs of these two species reported by others^29^, indicating an additional overpotential in our system.

### In situ electrochemical STXM of electrodeposition, electro-reduction and CO_2_ reduction catalysis by copper nanoparticles

**Fig. 4:**
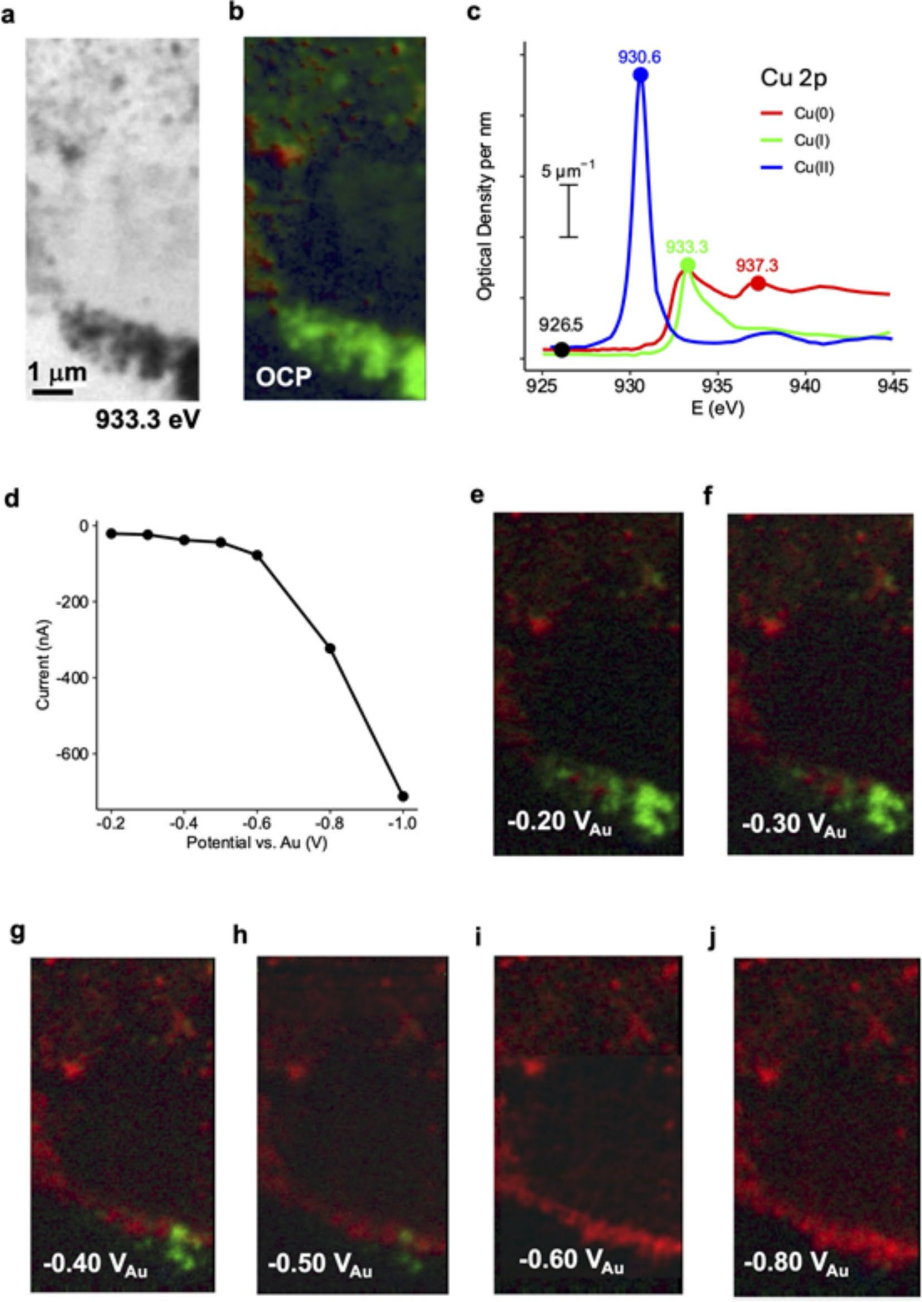
In situ flow electrochemical study of Cu catalyzed CO_2_ reduction. (**a**) Transmission image at 933.3 eV of electro-deposited Cu. (**b**) Color coded composite of Cu (red), Cu_2_O (green) and CuO (blue) component maps derived from a Cu 2p_3/2_ stack. (**c**) Reference spectra used in the fit^20^. The points indicate the energies used for the 4 energy short stack. (**d**) Current as a function of applied potential while measuring Cu 2p_3/2_ stacks. CO_2_ reduction commences at − 0.7 V_Au_. (**e**) through (**j**) color coded composite of Cu (red), Cu_2_O (green) and CuO (blue) component maps derived from stacks measured at the indicated potentials. Scale bar – see (**a**).

Figure 4 presents results from the use of the device for a 3-stage investigation of copper electrocatalysts under CO_2_R reaction conditions. This example shows the applicability of the device for complicated electrochemical experiments and energy applications. The goal of the study is to measure the oxidation state(s) of copper nanoparticles present under applied potentials where CO_2_R takes place. There is on-going debate in the literature as to the catalytically active species, with pure Cu metal, cuprous oxide (Cu_2_O), or subsurface oxygen being supported by different experimental and computational results^30^. The results presented here complement the in situ STXM^20,31^ and in situ spectro-ptychography^21^ results we have reported elsewhere.

First, particles were electro-deposited on the working electrode from a solution of 10 mM CuSO_4_ and 10 mM KCl using a short-CV protocol described elsewhere^20,21^. Figure 4a is a STXM image (at 933.3 eV) of the portion of the working electrode (WE) that was studied. Second, after preparing this sample, the electrolyte was changed to CO_2_-saturated 1.0 M KHCO_3_. Then a Cu 2p_3/2_ stack (925–948 eV, 40 points) was measured at open circuit potential (OCP). Figure 4b is the result of a fit of that Cu 2p_3/2_ stack to quantitative optical density (OD/nm) spectra of Cu, Cu_2_O and CuO (Fig. 4c). This analysis shows that the as-deposited Cu materials are mainly Cu_2_O, with some Cu metal. The spectral evidence for this is presented in Supplementary Fig. 5. In the third step, the applied potential was reduced to lower potentials in a step-wise manner while recording the current (chronoamperometry). A 4-energy Cu 2p_3/2_ stack (926.5, 930.3, 933.3 and 937.3 eV, at the points shown in Fig. 4c) was measured at each of 7 potentials. The recorded average current at each potential is plotted in Fig. 4d. The color-coded composites of the chemical mapping at each potential are shown as Fig. 4e and j. The Cu_2_O is completely reduced to metallic Cu by -0.6 V_Au_, which is still above the potential of the onset of the CO_2_ electro-reduction (− 0.7 V_Au_). Under CO_2_R conditions (V_Au_ < − 0.7) there is less than 1% Cu_2_O left according to a detailed in situ STXM study of Cu^20^. The results showed the real active catalyst for the CO_2_R reactions is Cu metal. Evidence that the current measured < − 0.7 V_Au_ is associated with CO_2_ reduction reactions generating reduced products (CO, CH_4_, C_2_H_4_, …), is presented elsewhere^20,32^.

### In situ electrochemical STXM of organic microbial biopolymers

**Fig. 5:**
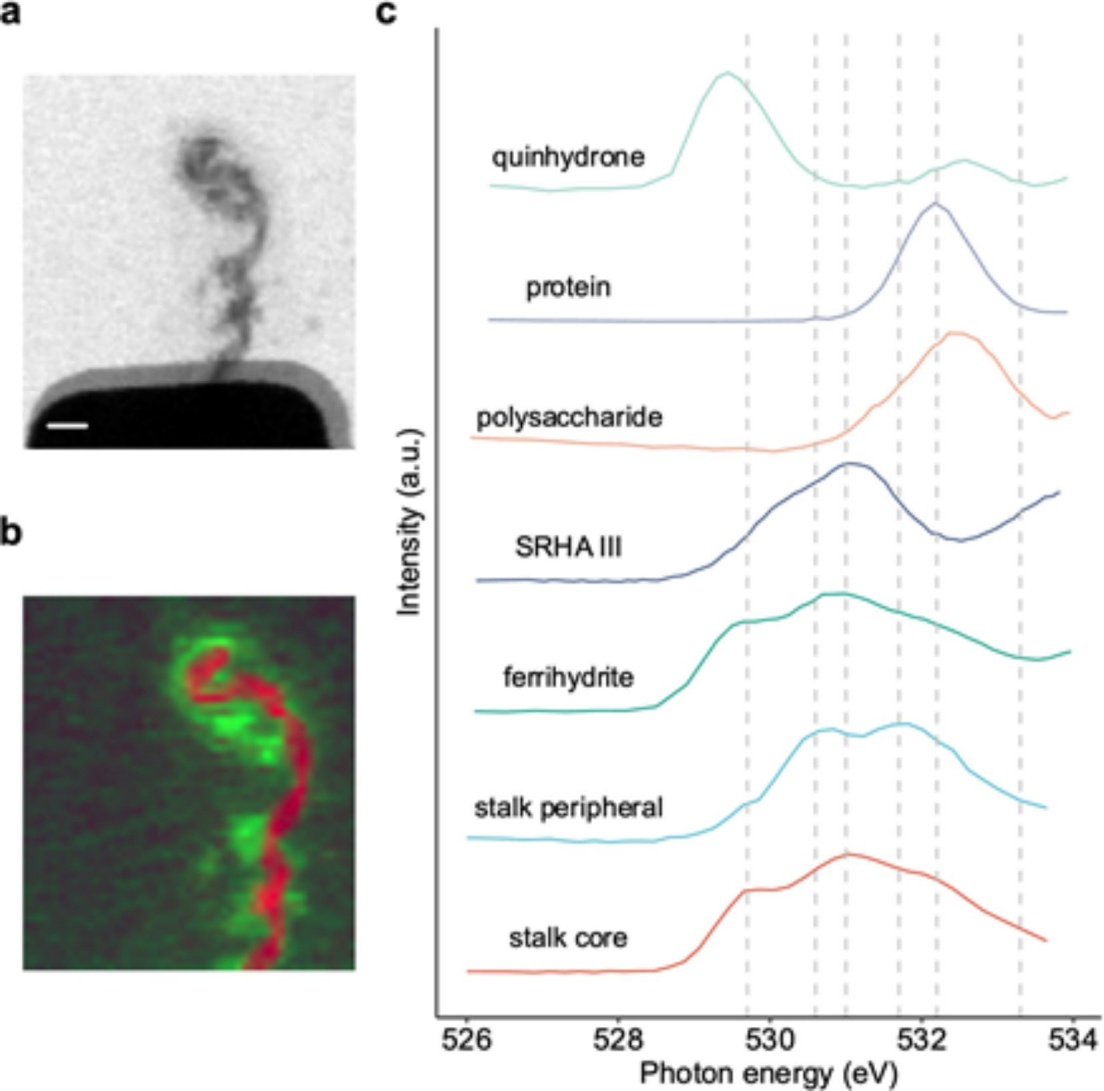
Twisted stalk placed on an electrode. (**a**) Transmission image of the twisted stalk placed on the electrode at 520 eV. (**b**) Component map showing regions of the sample with different spectral signature (core part in red and peripheral part in green). (**c**) Corresponding spectra compared to reference spectra of common organic compounds and iron minerals. Dashed lines indicate key features included as peaks in the fitting model and additional tail peak. (Scale bar: 2 μm).

As the next step of complexity, we used the device for spectro-electrochemical characterization of individual environmental particle samples. We probed the redox behavior of a microbial biopolymer structure called a twisted stalk, that was previously deposited onto the electrode using a micromanipulator. Twisted stalks are formed by e.g. microaerophilic Fe(II)-oxidizing bacteria. They consist of organic biopolymers that can complex Fe^3+^ ions and, at later stages, encrust in iron ox(yhydrox)ides^3^. Figure 5 shows the twisted stalk probed for this study, mounted on an electrode inside the electrochemical flow cell. O 1s NEXAFS spectromicroscopy of the hydrated stalk measured in 0.05 M phosphate buffer at pH 6 showed a heterogeneous composition of the twisted stalk, with the core of the stalk differing from the peripheral part (Fig. 5b-c). The strongest features in the O 1s spectrum of the stalk core were at 531.0 eV, with a shoulder at 529.7 eV. In contrast the stalk periphery had two equally prominent features at 530.6 eV and 531.7 eV. The peak at 531.0 eV was not present in the periphery.

**Fig. 6:**
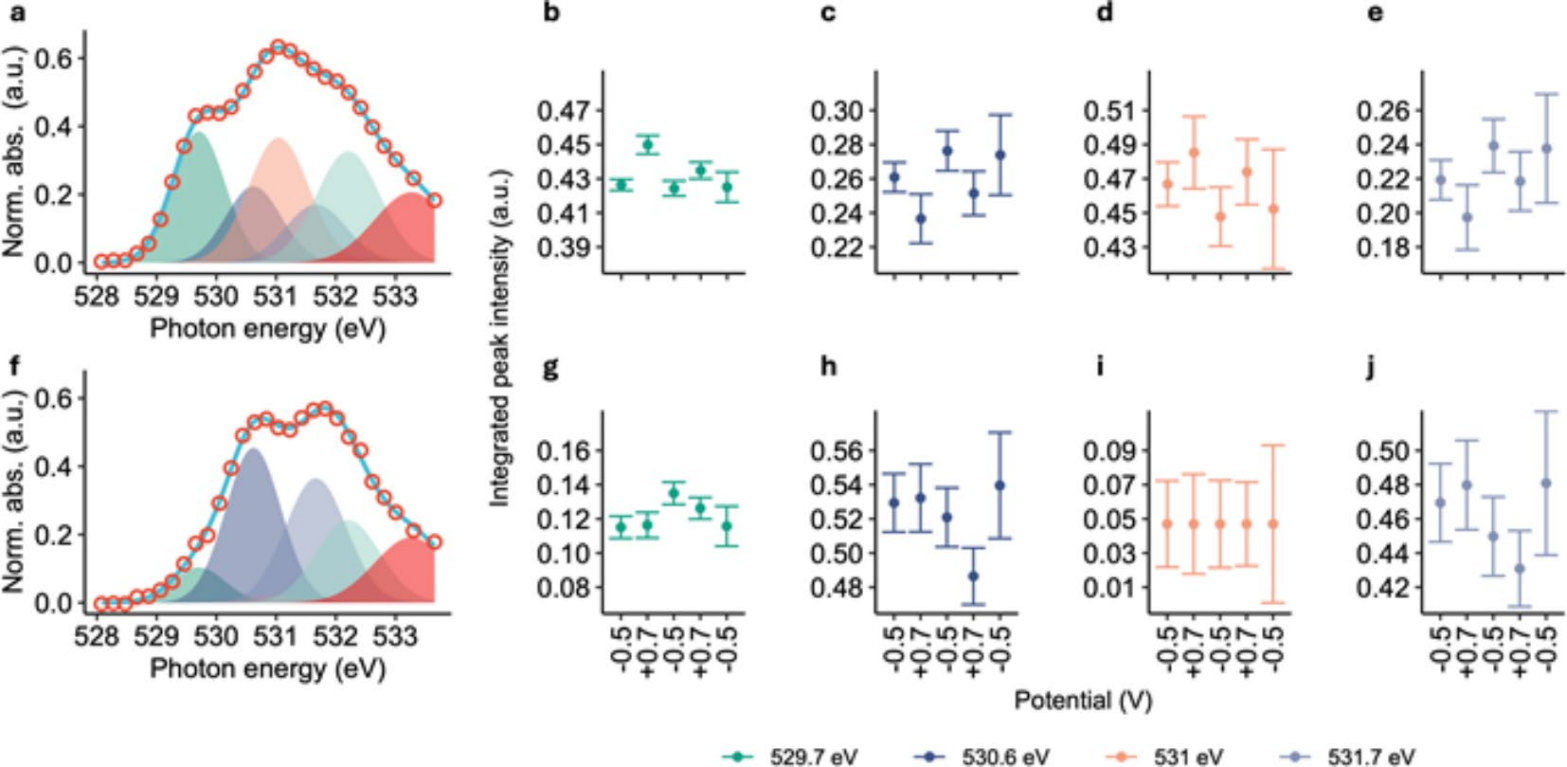
Deconvolution of stalk spectra. Measured spectrum (open circles) vs. fit (solid line) for the stalk core (**a**) and peripheral (**f**). The fit was composed of 6 Gaussian peaks. Fitted peak areas with respect to the applied potential vs. Au. (**b**–**e**) Core part of the twisted stalk. (**g**–**j**) Peripheral part of the twisted stalk.

Spectral decomposition was used to evaluate the response of individual peaks to the applied potential. Spectra extracted from both the core and peripheral regions of interest (ROIs) were analyzed with respect to the applied potentials (Fig. 6). Interestingly, the core and peripheral regions of the stalk behaved differently to the applied reducing or oxidizing potential. The twisted stalk core showed reversible reduction/oxidation indicated by a systematic response to the applied potentials of several peaks (Fig. 6b-e). Peaks at 529.7 eV and 531 eV increased in intensity, going from a negative to positive potential, whereas peaks 530.6 eV and 531.7 eV decreased with applying a positive potential. In contrast, the peripheral part did not show a systematic response to the applied potential. An intensity change correlated to the applied potential was not observed for any of these peaks. A reversible reduction/oxidation process did not occur.

## Discussion

In this study we have demonstrated various capabilities of our novel, modular, in situ flow electrochemical STXM platform for spectromicroscopic identification and electrochemical characterization of redox-active species on a sub-100 nm spatial scale. Specifically, we demonstrated both solution based in situ CV studies, and more complex in situ flow electrochemistry experiments, involving exchange of electrolyte solutions and in situ electrochemistry on individual particles.

### Capabilities of the novel, in situ flow electrochemistry STXM device

There is an increasing demand from various areas of science and technology for methods and approaches that allow for performing in situ electrochemistry experiments while at the same time acquiring spatially resolved chemical speciation. Whereas commercial systems for in situ transmission electron microscopy (e.g. – Hummingbird, ProtoChips, DENS Solutions, Norcada) have existed for over a decade, only a few devices for synchrotron-based STXM are available, and those have been introduced only in the last few years.

The earliest attempts to combine electrochemical sample characterization and manipulation with soft X-ray STXM used entirely sealed measurement cells made from a sandwich of two Si_3_N_4_ windows^8^. Once sealed, these cells did not allow for any electrolyte exchange. This concept was an important step in the course of the development of devices for in situ electrochemical STXM and was successfully used for polymer research^8^, electro-catalysis research^10^ and, for the first time, characterization of individual redox-active twisted stalks^11^. In many cases, however, electrochemical STXM experiments require a timed or continuous replacement of electrolytes or reactants, as relevant concentration ranges of the reactants and the volume inside the STXM measurement cell are very limited. Another serious limitation of a closed measurement cell is that any gas evolution during the electrochemical experiment, such as by accidental water splitting or degassing of an electrolyte, can destroy the device. In closed system devices, gas formation often results in a failure as these do not allow for the removal of gas bubbles that are trapped in the STXM transparent region. Overpressure from gas formation cannot be released from the cell, often resulting in breakage of the sensitive silicon nitride membranes that are typically less than 100 nm thin, thus fragile.

As a consequence of these challenges, the next step of development was an in situ flow-through electrochemistry device for STXM. Prabu et al. reported a 3d-printed design, and demonstrated its use for cyclical in situ Cu deposition onto a working electrode, followed by stripping^12^. This flow-through design, with one inlet and one outlet tube, was already much more flexible compared to the previously used static cells and allowed for higher electrochemical complexity such as CV experiments resulting in precipitation of a solid phase from a dissolved species. Major limitations of this design were the long time scales (> 1 h) that were required for a complete exchange of the electrolytes in the tubing system. Unfortunately, the material properties of the high-resolution 3D prints did not allow for the desired, high flow rates because of limited pressure resistance. This caused additional difficulties in the vacuum environment of a STXM. The flow rates achieved were limited to 0.8 µL min^− 1^. This limitation did not allow for efficient electrolyte exchanges during a STXM experiment. The same problem of relatively low flow rates also limits the versatility of several commercial measurement cells for in situ electrochemistry STXM.

To circumvent this limitation, our modular system was designed with two inlet and two outlet channels respectively that merge very close to the actual measurement volume (Fig. 2c). This allows for efficiently flushing the inlet tubing by using the second channel as a bypass to the measurement cell. As illustrated in Fig. 2, the relatively large diameter of the inlet tubing and the microfluidic channels in the PDMS allow for an efficient replacement of the electrolyte. Only the volume of the short connection between the junction of the inlet channels and the volume within the Si chip sandwich needs to be pushed through the actual measurement cell, which – with a water layer thickness of only a few µm – is the flow limiting resistance and the position of pressure emergence.

The design of the device presented here has several advantages:

The handling of the device was improved over the earlier 3D printed approach so that the assembly times of an experiment at the synchrotron were reduced by a factor of 5 from hour time scales to tens of minutes. Also, the risk of leakage in the vacuum environment of the STXM is minimized by the assembly, as both the plasma bonding between PDMS and glass as well as between PDMS and the Si chip, and the epoxy sealing of the Si chip sandwich have been shown to reliably withstand pressure differences of several atmospheres. Flow rates as high as 2µL.m^− 1^ through the chip area can be established without difficulty, whereas the earlier concept based on O-ring seals in a 3D printed device could only be operated up to flow rates of 0.8 µL.m^− 1^ without leakage. The faster flow rates could ameliorate the diffusion limit due to the very thin electrolyte layer (a few µm). When the electrochemical reactions are occurring rapidly, the neutral or ionic reactants in the thin electrolyte layer can be completely consumed. At the same time product species may be inefficiently removed from the thin electrolyte layer around the working electrode surface into the chamber exit channel, providing mass transfer problems. The liquid-flow system in our device allows electrolytes to flow at a controlled fast flow rate which can improve the mass transfer to a degree and show a better electrochemical performance. However, the diffusion limit is still expected to affect the electrochemical reactions, slowing down the oxidation and reduction processes, thus providing a fairly large potential difference (overpotentials in anodic peaks) between the redox peaks in the fast CV scanning (Fig. 3). This challenge exists for all in situ systems and devices with thin electrolyte layers; we are still trying to solve this problem.

The PDMS channel design of the device presented here allows for more complex experiments, for example, electrolyte changes while maintaining in situ conditions. This is relevant for experiments where oxidation and/or reduction is to be compared with and without a mediator or electron shuttle. Earlier concepts with only 1 inlet and outlet channel respectively, required hours of flushing for a single electrolyte change, which is not an efficient use of limited synchrotron measurement time. The time required for electrolyte exchange (currently in the range of 2 min) can be further decreased by reducing the cross-section of the liquid channels in the PDMS layer, which are currently 100 μm x 200 µm. Time scales in the range of tens of seconds are feasible without increasing the required pressure.

The modular and flexible design of our device with microfluidics prepared from PDMS allows for easy implementation of microfluidic mixing channels or further changes in the number of inlet/outlet channels. In case of chemical incompatibility of PDMS with the required electrolyte, other materials such as polycarbonate or other polymers can be used instead of PDMS and can be connected to the glass and to the silicon chip by plasma-bonding. However, this process needs to be optimized. Vacuum and/or pressure tests are necessary to avoid leakage during in-vacuum applications.

The device presented here is very cost efficient, in particular considering the modularity. Since the fluidic chip with the electrochemical cell can be prepared individually and loaded into a mounting frame within a few minutes, only one or two mounting frames are required to use synchrotron beamtime efficiently.

Due to its entirely flat front side, the device can be operated safely in typical soft X-ray STXM setups down to the C 1s absorption edge, where the distance between the order sorting aperture and the focal point of the zoneplate can be as small as 300 μm or even less. Of this free working distance, 200 μm are “used” by the thickness of the Si-chip that holds the top Si_3_N_4_ window (100 μm capping windows also exist), so that the front of the device will be in very close proximity to the aperture. Therefore, the assembly of the device needs to be done carefully.

The presented device is compatible with the conventional 3-pin kinematic mounting system that most synchrotron-based STXM instruments are equipped with. However, due to its modularity, the device can be adjusted easily for different geometries in different STXM environments. Additionally it is in principle also compatible with other analytical microscopy approaches such as in situ Raman or in situ FTIR microscopy studies^33^.

### Electrochemical characterization of Fe(CN)_6_^3+^ model system

Despite some small discrepancies, the Fe L_3,2_ spectra measured (Fig. 3a and b) are in agreement with literature spectra^15,23,24^. The close similarity of the spectra of the solution species (Fe(CN)_6_^n−^, *n* = 3, 4) to that of the solid indicates the immediate environment of the iron ion is not changed by dissolution. In each case, the Fe ion is co-ordinated by six cyanide ligands^24^. The considerable differences between the spectra of these two species, in particular the 706.2 eV peak that is only present in K_3_[Fe(CN)_6_], are sufficient to easily differentiate these two species. The anodic “oxidation” and cathodic “reduction” processes in this system can be expressed as^34,35^:

2 $$[{\text{Fe}}({\text{CN}})_{6} ]^{{3 - }} + {\text{e}}^{ - } \rightleftarrows {\text{ }}[{\text{Fe}}({\text{CN}})_{6} ]^{{4 - }}$$

During the CV of [Fe(CN)_6_]^4−^ the anodic potential reached a sufficiently positive value (+ 0.05 V_Au_) to oxidize the solution species to [Fe(CN)_6_]^3−^. When the scan direction reverses, the [Fe(CN)_6_]^3−^ is reduced to [Fe(CN)_6_]^4−^ as indicated by the sharp reduction peak at 0 V_Au_. As expected, there was no oxidation peak in the first cycle when the CV of [Fe(CN)_6_]^4−^ was scanned in the oxidative direction, starting at 0 V_Au_, (see Fig. 3b). However, after switching the scan direction and scanning to − 0.2 V_Au_ a sharp reduction peak was observed, corresponding to the reduction of [Fe(CN)_6_]^3−^ to [Fe(CN)_6_]^4−^. On the second, and subsequent cycles the oxidation peak was observed at -0.07 V_Au_. The peak anodic current in the CV of [Fe(CN)_6_]^4−^ was much lower than that in the CV of [Fe(CN)_6_]^3−^. This lower current may be due to slower migration to the positively charged working electrode of [Fe(CN)_6_]^3−^ as compared to [Fe(CN)_6_]^36^.

Rooney et al.^36^ showed that, when studying very dilute ferric/ferrocyanide solutions (0.5 mM) without additional electrolyte, the ferric/ferrocyanide system using Au electrodes is not reversible, and there is about 0.1 V difference in the mid-point of the CVs for the ferric and ferrous cyanides, just as we have observed. However, when 0.1 M KCl was added as a supporting electrolyte, the mid-points of the CVs for the ferric and ferrous cyanides are aligned and the curves are as expected from a reversible couple.

Rosendahl^37^ used a flow electrochemical STXM cell to study solution electrochemistry of FeSO_4_. They observed a spatial gradient of Fe(II) and Fe(III) species between the WE and counter electrode (CE). Although we had expected a similar behavior in the present measurements, in fact the electrochemical state of the solution was always uniform. (Note the STXM imaging results measured during the CV study (Fig. 3B) are reported elsewhere^38^.)

Despite the technical challenges, X-ray absorption spectroscopy of liquids is a growing field. It includes studies of pure liquids and liquid phase chemical reactions^39^ as well as studies of the interface of liquids and solids^40–42^ The latter are particularly important since chemistry at solution – electrode interfaces is critical for many technologically important processes that involve electron transport across such an interface, including biogeochemical systems, batteries and electrocatalysis. The ability of this in situ device to measure XAS very close to the electrode surface (with ptychography, potentially within 10 nm laterally) with full control of the electrode potential and thus electric field, offers the potential for detailed studies of electric double layers^43,44^, and other phenomena.

### STXM of in situ electrodeposition, electro-reduction, and CO_2_ reduction catalysis by copper nanoparticles

In the first step of this study, Cu nanoparticles were electrodeposited in situ from a 10 mM CuSO_4_ and 10 mM KCl aqueous solution, In the second step, the electrolyte was changed to CO_2_ saturated KHCO_3_ aqueous electrolyte using the rapid electrolyte exchange capabilities of this device. In order to produce highly efficient catalyst particles on the WE, the initial Cu^2+^ solution needs to be replaced rapidly; otherwise, the catalyst particles dissolve within a few tens of minutes. In the third step, the quantitative speciation capabilities of STXM were used to map the morphology and oxidation states of the catalyst particles as a function of applied potential. The results (Fig. 4) show that the morphology of the Cu catalysts remained similar to that just after changing the electrolyte, but the chemical composition changed as the potential was reduced from − 0.2 to − 0.6 V_Au_. The evolving oxidation state of Cu can be seen easily from the color composite maps of Cu (red), Cu_2_O (green) and CuO (blue) at different applied potentials (Fig. 4e-j). The in situ results show that the initially electrodeposited Cu_2_O particles are not stable. The Cu_2_O is converted to metallic Cu at potentials between − 0.2 to − 0.6 V_Au_, prior to the onset of the CO_2_R reaction at − 0.7 V_Au_^20^. This, along with the results at still lower potential, indicates that metallic Cu is the active oxidation state for CO_2_ reduction catalysis. Moreover, the ability to conduct in situ STXM at the C 1s edge using this device was used to confirm that CO_2_R reactions were occurring by measuring the signal of the reactant (CO_2_) and products (CO, alkenes and alcohols)^32^. This allows for product identification and, ultimately, quantification to evaluate selectivity and efficiency under reaction conditions. These results will improve our understanding of CO_2_R electrocatalysts, hopefully leading to better catalysts.

### Electrochemical characterization of twisted stalks

Here we demonstrated the reversible oxidation and reduction of redox-active components of twisted stalks. These are complex structures, composed of organic molecules, mainly polysaccharides, and iron ox(yhydrox)ides that precipitate upon oxidation of dissolved Fe^2+^ ions by the microaerophilic bacteria and subsequent complexation of the Fe^3+^ ions within the organic structure^3,45^. To our knowledge, only one other study of the redox properties of twisted stalks has been reported. That study, conducted at the C 1s absorption edge using this device^11^, demonstrated the reduction of quinone moieties. In this study we chose to focus on the O 1s absorption edge to gain insight into the composition of, and the interaction between, the organic and the Fe-mineral phases. The O 1s pre-edge region (528–532 eV) is below the onset of the water absorption and hosts peaks for both organic phases (carbonyl functional groups, such as quinones and carboxyls)^33,35^ and Fe-mineral phases (e.g. Fe-ox(yhydrox)ides)^42,46,47^ (Fig. 5c). The spectral features in the pre-edge region of O1s allowed for distinguishing between the individual components of the twisted stalk such as organic matter and Fe-oxyhydroxides. At the same time, the electrolyte thickness of the sample (~ 4 μm) resulted in a high absorbance both above the O 1s absorption edge and at the C 1s edge preventing measurements at the Fe2p or C1s absorption edges. Due to the thickness of the twisted stalk itself (see Fig. 5a), this problem cannot be solved for this particular sample.

Our results clearly showed redox activity in the core of the twisted stalk, whereas redox inactive regions were identified in the peripheral part, revealing the complex nature of these structures on the micron scale. In the core region of the stalk, peaks at several energies were redox sensitive in reverse directions (i.e. their intensity increased or decreased upon reduction and decreased or increased upon oxidation). At first glance, the stalk core spectrum contains features that are very similar to that of ferrihydrite (Fig. 5c). This occurrence of ferrihydrite is expected as the organic polymers composing the twisted stalks are known to act as templates for the nucleation, precipitation and eventually recrystallization of Fe-ox(yhydrox)ides such as ferrihydrite as a phase of low crystallinity or the more crystalline phase lepidocrocite^2^. However, in contrast to the spectral interpretation by Chan et al.^2^, we postulate a small but significant contribution of the organic carbon to the absorbance at the O 1s absorption edge. In an earlier 3D STXM tomography study of a twisted stalk at a similar stage of encrustation, we measured edge-jumps at the C 1s and Fe 2p absorption edges of similar heights^48^. Interpolating both the contribution of an organic polymer such as xanthan and the contribution of ferrihydrite in this ratio to the O 1s absorption edge allows for roughly estimating a contribution of the organic structure of up to 20% in the pre-edge region of the O1s absorption edge (see further explanation in Supplementary Fig. 6).

Thus, we interpret the major fraction of the peak at 529.7 eV and the peak to derive from a Fe-oxyhydroxide phase of low crystallinity. The peak energies match those of the main features of ferrihydrite or lepidocrocite well^2^. The more crystalline Fe-oxide hematite has a double peak feature in the pre-edge of O 1s with an energy difference of 1.3 eV, which in general would also fit^42,49^. However, hematite is not formed under the environmental conditions where our bacteria grow and hematite has - in contrast to ferrihydrite and lepidocrocite - never been reported in association with twisted stalks. Additionally, reversibility of reduction and oxidation resulting in the rapid dissolution and rapid precipitation of the highly crystalline phase hematite is very unlikely. In contrast, a reversible dissolution and precipitation of a low crystallinity ferrihydrite phase that is strongly associated with the organic backbone of the stalk seems to be a much more realistic mechanistic explanation for our observations. The fact that peaks 1 and 3 only vary in intensity by ~ 6% and ~ 9% respectively, but do not fully disappear, indicate an incomplete reduction at the applied potential. The dissolved Fe^2+^ ions resulting from this reduction must, to a large extent, be retained within the core by adsorption. Otherwise, we would expect the peaks 1 and 3 to contribute less to the spectrum with each potential cycle because of diffusive losses of Fe^2+^. We do observe such loss, but it is not significant. The adsorption that we postulate here would require the presence of negatively charged functional groups within the core region of the stalk. Interestingly, the change in the 530.6 eV peak (which contributes significantly to the main feature in the absorption spectrum of ferrihydrite) upon potential change is reversed compared to that of the 529.7 eV peak. This indicates that the reduction and oxidation ferrihydrite cannot explain the observed variations by itself, but the contribution of another redox-active moiety is required to fully explain the observed changes.

In contrast to the spectrum of the core, the spectrum of the peripheral part of the stalk does not exhibit peak 3 and the contribution of peak 1 is considerably smaller. This indicates that ferrihydrite is not precipitated within the peripheral part of the stalk, but only in the core, at least at this particular stage of stalk formation. Additionally, the small but obvious contribution of peak 1 to the spectrum extracted from the smear in the periphery indicates that not only ferrihydrite is contributing to the absorption at this energy, but also organic functional groups.

Organic functional groups such as carbonyls and carboxyls show features at the O1s pre-edge. The O 1s → π*_C=O_ resonance of carbonyls are found at relatively low energies between 529 eV and 533 eV, depending on their chemical environment^50^. In quinones this resonance is generally lower as compared to other functional groups^47^, which can also be seen in our measurements of quinhydrone, which is a mixture of hydroquinone and benzoquinone.

A certain contribution of quinone moieties to peak 1 at 529.7 eV is likely from our observations, but an unambiguous and quantitative calculation of this contribution is currently not possible as the respective associated phenolic moieties that should increase upon reduction, absorb at X-ray energies beyond the onset of the water absorption^47^ and thus cannot be identified or quantified in this experiment. However, the larger reversible variations of the peak at 529.7 eV upon reduction and oxidation, in comparison to the sum of variations of the peaks underneath the main feature of ferrihydrite (530.6 eV and 531.0 eV) are clear indications that this peak cannot entirely originate from the Fe-oxyhydroxide but must originate at least to some extent from the organic fraction of the twisted stalk, namely the quinone moieties.

Since we assign peak 1 at 529.7 eV in the spectrum extracted from the periphery to quinone moieties, we should in principle also expect a certain redox sensitivity. The fact that we did not observe this indicates the applied potential was not low enough to reduce the quinone moieties in the periphery. This could either be explained by a different organo-chemical structure of the periphery in comparison to the core of the stalk, resulting in a different potential range of redox activity, or by an interaction between the organic functional groups and the Fe in the ferrihydrite phase is required to render the organics redox-sensitive in the applied potential range.

Carboxyl functional groups have a strong O 1s →π* pre-edge feature at positions ranging from 530.6 to 531 eV^46^. Our measurements of the reference Suwannee River Humic Acid that is rich in carboxyl and quinone groups^51^, shows a broad feature with a peak at 532.2 eV and a shoulder at 531.3 eV. The presence of these features indicates there are negatively charged functional moieties in the core of the stalk. This is in agreement with the previously discussed adsorption of Fe^2+^ ions deriving from the partial reductive dissolution of the ferrihydrite phase in the core of the twisted stalk.

The observed, distinct redox behavior of the organic and inorganic constituents mark one step further in understanding the fundamental properties of twisted stalks and help to further our understanding of the mechanism of Fe(II)-oxidation by microaerophilic, stalk-forming, Fe(II)-oxidizing bacteria. The novel in situ electrochemical device will be integral to achieve multi-edge analyses required to identify and to quantify the involved organic/mineral fractions further.

An unambiguous and precise quantification of the individual contributions of quinone moieties in the organic backbone of the twisted stalk and those of associated Fe-ox(yhydrox)ides to the variations of peak areas upon reduction and oxidation, is currently not possible. Additional measurements at the C 1s and Fe 2p absorption edges are highly desirable. Unfortunately, these could not be achieved in this project because of the limited transparency at these edges due to the thickness of the water layer in this sample, which is dictated by the intrinsic thickness of the twisted stalk itself.

## Conclusions

In this study we present the development, implementation, and functionality of a novel in situ electrochemistry STXM device that allows for experiments combining electrochemical manipulation of samples with the X-ray spectromicroscopic characterization at the 10s of nm spatial scale. We demonstrated the capabilities of the novel device in various applications of electrochemistry with an increasing level of experimental complexity:

- in the liquid phase.
- electrodeposition of catalyst particles from the liquid phase.
- in situ characterization of electrodeposited catalyst particles.
- electrochemical characterization of twisted stalks with heterogeneity on the submicron scale.

This opens up a plethora of experimental possibilities in various fields of material and environmental sciences including energy storage, catalyst research and biogeochemistry.

## Methods

### Modularin situ flow electrochemistry platform

The frame piece of the in situ electrochemistry platform was CNC (computer numerical control) milled from a copper clad FR-4 glass epoxy laminate of 2 mm thickness. A 4-pin cable connector and CuBe_2_ contact strips for contacting the electrode chip were soldered onto this frame. The retaining piece was CNC milled from a 1 mm thick polycarbonate sheet and the screw holes were threaded using an M1.6 tap.

The microfluidic chip component is a two layered setup comprised of a PDMS (poly dimethyl siloxane) layer housing the microfluidic channels, plasma bonded to a glass layer, sealing the channels. It was fabricated following common protocols for PDMS microfluidic devices^52^. A master mold for the PDMS layer was made by photolithography. SU-8 2075 photoresist (MicroChem) was used according to the manufacturers’ protocol to achieve a final channel height of 100 μm. Sylgard 184 (Dow Corning) was mixed in the ratio 1:10 (curing agent: siloxane) and poured in the mold to a thickness of 1 mm. Curing was performed at 60 °C for 2 h. The cured PDMS piece was cut to dimensions and holes for fluidic connections were punched using a 0.75 mm diameter biopsy punch. Pieces of silicone tubing (ID 1 mm, 2 cm length) were attached to the four inlet/outlet holes using silicone glue (Elastosil E43, Wacker, Germany). This bond was allowed to cure for 24 h at RT. Subsequently, the part was bonded to a glass cover slip (Menzel, Germany) by plasma bonding inside a plasma oven (13.56 MHz, 30 W, Diener Electronic, Germany) in an air plasma at 300 mTorr. Exposure times were 120 s for glass and 15 s for PDMS. After bonding the glass was cut to dimension with a diamond-cutting tool.

Detailed information about the silicon/silicon nitride electrochemistry cell, used in this study, is published by Prabu et al.^12^. The electrode chip was bonded to the PDMS surface of the fluidic chip using plasma bonding as described above. The spacer chip was placed and aligned on the electrode chip. The edges of this assembly were sealed using Torr Seal (Agilent Technologies), ensuring no epoxy resin was caught between the two chips or protruded above the outer surface of the spacer chip.

### Leakage test

Leakage tests were performed by filling a fully assembled device with aqueous brilliant blue solution and sealing off three of the four channels. Channels were sealed using Silicone Glue (Elastosil E43, Wacker, Germany). A syringe pump (Cetoni) was used to introduce ink solution through the remaining channel. The pump was connected via an FEP tube (o.d. 1.58 mm, i.d. 0.5 mm) inserted into the silicone tube connector of the fluidic chip. Hydraulic pressure was built up by continuously pumping solution. The pressure was recorded using a pressure sensor (Honeywell 24PCGFM6G, Honeywell Inc., USA). Additionally, the device was tested under vacuum conditions inside the STXM tank of the SoftiMAX beamline at MaxIV laboratory. A pressure of 0.029 mbar was reached after 20 h of pumping with a roughing pump without any leakages (Supplementary Fig. 2).

Fluid dynamics were monitored via laser scanning microscopy, measuring the fluorescence intensity of two different fluorescence indicators inside the electrochemistry cell over time. Aqueous solutions of calcein and resorufin (Sigma Aldrich) were used. Each solution was connected to separate inlet ports of the in situ device. The flow of each dye was controlled independently by a multichannel syringe pump (Nemesys, Cetoni GmbH, Korbussen, Germany). In the beginning the in situ system was filled with the resorufin solution. The flow through the cell was switched from one channel to the other several times, while recording the fluorescence intensities of the two dyes. Fluorescence intensities were sampled in intervals of 3.5 s. For each timepoint the average fluorescence intensity over the window area was taken.

### STXM in situ electrochemistry

The in situ electrochemical STXM experiments were conducted using the ambient-STXM on CLS beamline 10ID1^53^, and the Maxymus STXM on Bessy-II UE46 beamline, in each case, using a zone plate of 40 nm outermost zone width. A portable potentiostat (either a PocketStat, Ivium Technologies, Eindhoven, Netherlands or an EMStat 4 (Palmsense) was used for the electrochemical experiments.

### STXM electrochemistry of hexacyanoferrate

Potassium ferricyanide (K_3_[Fe(CN)_6_]), (≥ 99.0% ) and potassium ferrocyanide (K_4_[Fe(CN)_6_].3H_2_O, (≥ 99.95%) were purchased from Sigma Aldrich and used without further purification. 0.1 M aqueous solutions were prepared using deionized water.

The device was first filled with 0.1 M potassium ferrocyanide and the Fe L_3,2_ spectrum was measured. Subsequently the electrolyte was changed to 0.1 M potassium ferricyanide using the bypass feature of the in situ platform. Electrolyte exchange was verified by acquiring image stacks before and after solution exchange. CV was performed for each of the potassium ferro- and ferricyanide solutions. The CVs were conducted in a potential range of + 0.3 to -0.6 V vs. Au (V_Au_) with a scan rate of 20 mVs^− 1^. During the CV measurements a constant electrolyte flow of 15µL h^− 1^ was maintained using a syringe pump (NE-1010). Several Fe L_3,2_ stacks, consisting of 160 images over the photon energy range of 700 to 740 eV, were measured. STXM data was analyzed using aXis2000 software^54^.

### STXM electrochemistry of CO_2_R experiments

The experimental details for in situ STXM measurements of CO_2_R reactions using this device have been reported elsewhere^20^. Briefly, copper nanoparticles were electro-deposited from a solution of 5 mM CuSO_4_ and 5 mM KCl on to the Au working electrode using 3 CV cycles (potential scanned at a rate of 20 mV s^− 1^ between 0 and − 0.4 V_Au_. The solution was then changed to CO_2_ saturated 0.1 M KHCO_3_ and then full Cu 2p stacks (40 energies from 925 to 948 eV) or 4-energy quick stacks were measured as the potential was step-wise changed from − 0.2 V_Au_ to − 1.0 V_Au_. Based on ex situ electrochemical measurements, in the CO_2_ saturated 0.1 M KHCO_3_ V_RHE_ = V_Au_ +0.4 eV.

### STXM electrochemistry of organic microbial biopolymers

### Sample preparation

Twisted stalks were sampled from an environmental biofilm containing iron oxidizing bacteria. The biofilm was located at a little trench, draining a forested wetland (50°08’05.78”N, 11°52’14.90”E). Individual twisted stalks were selected under a microscope using glass micropipettes on a Micromanipulator (TransferMan, Eppendorf AG, Hamburg, Germany) and carefully placed on electrode chip, prior to sealing the cell. O 1s reference spectra of the pure compounds were used for comparison. (Supplementary Table 2).

### Solutions

Due to the sensitivity of Au-electrodes towards Cl^−^ ions, 0.05 M phosphate buffer (monosodium phosphate, NaH_2_PO_4_, ≥ 99%; disodium phosphate, Na_2_HPO_4_·7H_2_O, ≥ 99.99%; Sigma Aldrich) of pH7 was used as an electrolyte. Solutions were prepared with ultra-pure water (resistivity > 18.2 MΩ cm), which was deoxygenated by boiling and purging with Argon for 1 h. The solutions were kept under oxygen free conditions until the beginning of the experiments. 2,2′-Azino-bis(3-ethylbenzothiazoline-6-sulfonic acid) diammonium salt (ABTS, ≥ 98%, Roche) was added to a final concentration of 0.005 M.

### Scanning transmission X-Ray Microscopy

Image stacks were recorded with pixel dimensions of 250 × 250 nm in the O 1s pre-edge region between 524 eV and 534 eV with an energy step size of 0.1 eV in the energy region of interest. The selected energy region includes absorption peaks specific for iron and quinone species. Above 534 eV, absorption saturation occurred due to the ~ 4 μm thick water layer, caused by the intrinsic thickness of the twisted stalk. Energy calibration was done using the O 1s → π* absorption peak of O_2_ at 530.8 eV^55,56^.

### Electrochemistry

After manually flushing the electrolyte individually with a syringe through the inlet and outlet channels using the bypasses, the in situ electrochemistry platform was completely filled with the electrolyte solution using a syringe pump outside of the STXM tank that was connected to the setup and adjusted to a flow rate of 18 µL h^− 1^ while the bypass channels were closed. Potentials were applied in chronoamperometric mode 20 s prior to the start of image or image sequence (stack) acquisition and kept constant until the acquisitions were finished. For each subsequent stack the potential was altered between − 0.5 V_Au_ and + 0.7 V_Au_. These potentials were chosen based on cyclic voltammograms of benzoquinone vs. Au. At the beginning, the system was preconditioned at + 0.7 V_Au_ for 3 min. The electrolyte flow was kept constant over the course of the experiments.

### Data analysis

aXis2000^54^ was used for image stack alignment, conversion to optical density (OD), and extraction of selected-area NEXAFS spectra. Transmission image stacks were converted to OD stacks based on the Beer-Lambert Law, OD = − ln(I/I_0_), where I is the photon flux transmitted through the sample region of interest (ROI), and I_0_ is the incident flux measured through the electrolyte and silicon nitride windows in an empty area adjacent to the area of interest.

Spectra of the same ROI of the twisted stalk were extracted from the image stacks at each potential step. These spectra were decomposed into individual peaks, allowing to detect changes of individual moieties of the stalk. Peak fitting of the spectra using Gaussian peaks was done using Athena^57^. The near edge X-ray absorption fine structure (NEXAFS) spectra of both the core area and the peripheral part of the stalk could be fit adequately using the same model with a minimum of five “analytical” peaks. The peak positions were chosen based on the energies of obvious features in the recorded NEXAFS spectra of both the core and the periphery (see dashed lines in Fig. 5). One additional Gaussian peak was required for modelling the “tails” of the pre-edge spectra. The model to ultimately fit the peak heights and areas for all spectral components was derived using an iterative process. Initially the peak energies were set to the positions of the features found in the recorded spectra. Peak widths (sigma values) were initially set to 0.5 eV. In a first iteration, the peak heights were fit by minimizing the models’ error. In a second and third iteration, only the sigma values or energies were optimized, respectively. This process was repeated until the models’ error was minimal.

The derived model was used to fit both core and periphery of the stalk. If a peak was not present, the contribution to the fit was close to 0, as for example in case of the 531.0 eV peak in the spectrum of the peripheral part of the twisted stalk. In that case it was manually fixed to 0 for the final fit.

## Electronic supplementary material

Below is the link to the electronic supplementary material.

Supplementary Material 1

Supplementary Material 2

## Data Availability

The datasets generated during and/or analyzed during the current study are available from the corresponding author on reasonable request.
