# Supplementary material for: Soft X-ray spectromicroscopic proof of a reversible oxidation/reduction of microbial biofilm structures using a novel microfluidic in situ electrochemical device
Source: Sci Rep. 2024 Oct 14;14:24009. doi: 10.1038/s41598-024-74768-9 (PMC11473636; doi:10.1038/s41598-024-74768-9)
Supplement: Supplementary file 2 — Supplementary Material 2 [file 41598_2024_74768_MOESM2_ESM.pdf]

Supplementary Information

Soft X-ray spectromicroscopic proof of a reversible oxidation/reduction of individual microbial biofilm structures using a novel microfluidic *in situ* electrochemical device.

Pablo Ingino[1], Haytham Eraky[2], Chunyang Zhang[2,3], Adam P. Hitchcock[2], Martin Obst[1*]

[1] Experimental Biogeochemistry, BayCEER, University of Bayreuth, D-95448 Bayreuth, Germany
[2] Chemistry & Chemical Biology, McMaster University, Hamilton, ON, Canada.
[3] Chemical Engineering, McMaster University, Hamilton, ON, Canada

[*]corresponding author: Martin.Obst@uni-bayreuth.de

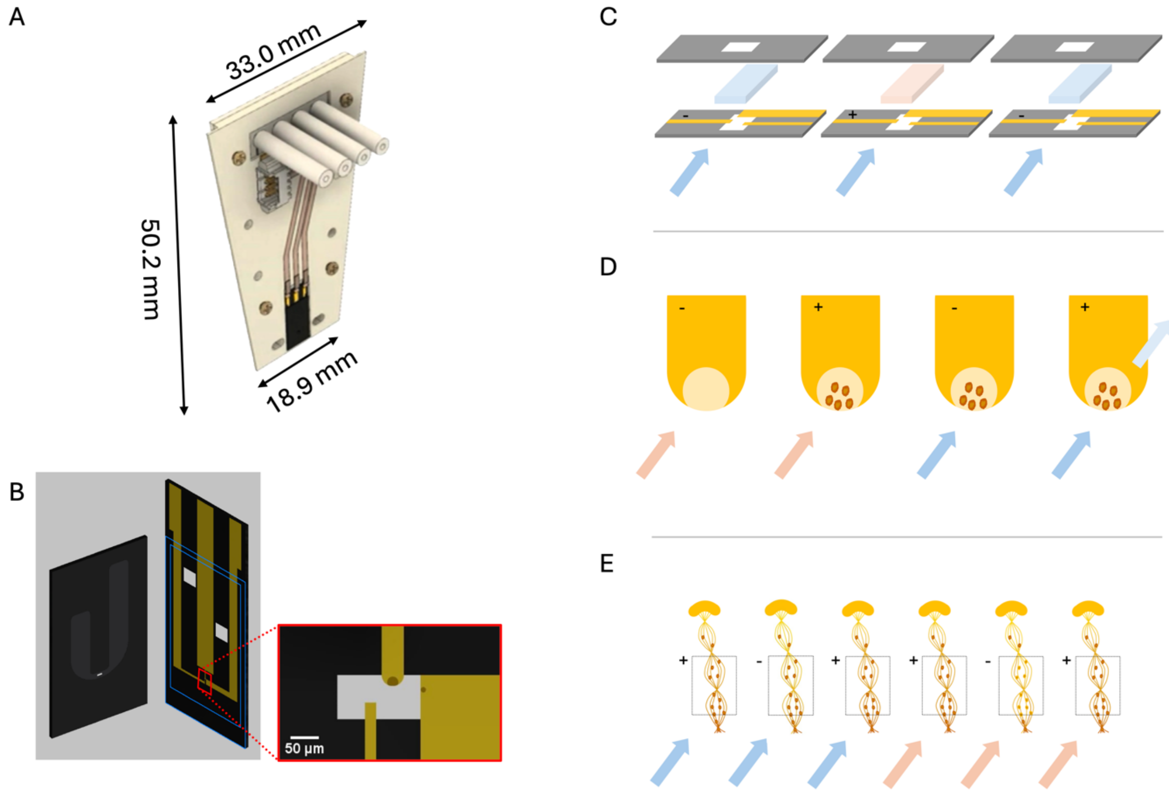

Supplementary Fig. 1. (A) STXM electrochemical device (front view) including dimensions. (B) Si chips with Si_3N_4 windows; top window with microfluidic channels; bottom window with 3 gold electrodes. (C to E) arrows indicate electrolyte flow while arrow colors indicate different electrolytes. + and – symbols indicate different potentials. (C) Sketch of electrochemistry STXM experiment in solution phase; dissolved species are oxidized/reduced depending on the potential while soft X-ray absorption spectra (XAS) of the dissolved chemical species in a micrometer thin water layer are measured through the Si_3N_4 windows. Depending on the chemical species of interest, this setup works best at high concentrations because of the limited absorption by the ions in solution. This setup refers to the presented example of ferri/ferrous cyanide. (D) Sketch of an electrochemistry experiment wherein a phase is electrodeposited onto the electrode. The electrodeposited particles can be analyzed by XAS. After an electrolyte change, the deposited material is used to catalyze another process. This sketch refers to the presented example of copper nanoparticles used as CO_2 reduction electrocatalysts. (E) A section of an individual twisted stalk is deposited with a micromanipulator onto the gold electrode. The redox activity of the complex sample composed of microbial polymers and iron oxyhydroxides is studied by XAS with various electrolytes. This sketch refers to the example of a twisted stalk of a microaerophilic Fe(II)-oxidizing bacterium.

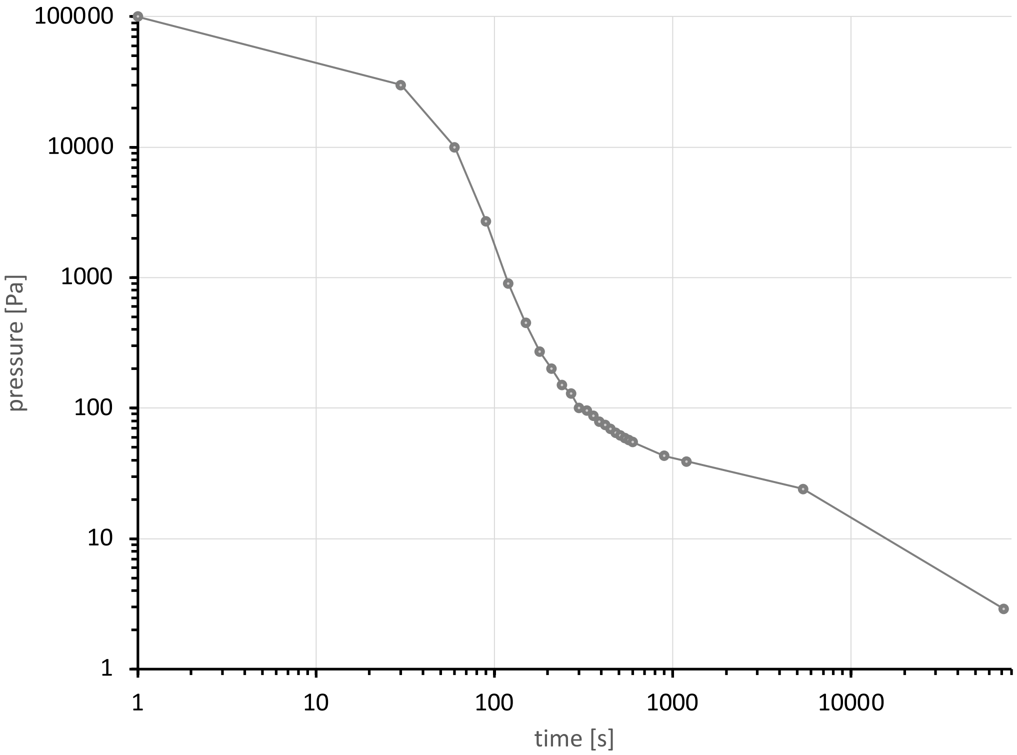

Supplementary Fig. 2. STXM tank pressure during *in situ* flow through experiment. The pressure was constantly decreasing due to pumping with a roughing pump. No leakage of the aqueous liquid into the vacuum occurred for the entire duration of 20 hours.

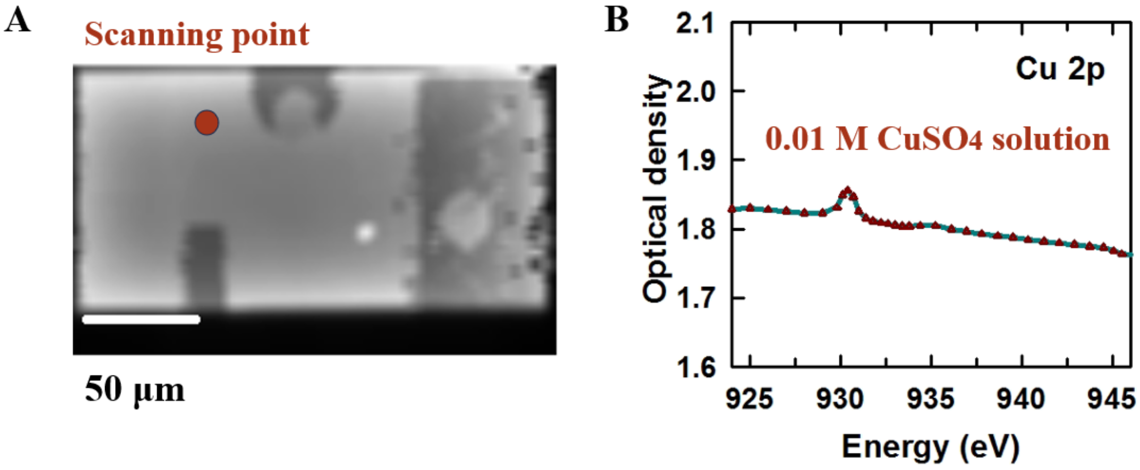

Supplementary Fig. 3. The Cu 2p spectrum of a ~ 2 µm thick 0.01M ($CuSO_4$+KCl) electrolyte layer. (a) STXM transmission image at 933 eV. The dark red dot indicates where the electrolyte spectrum was measured. (b) The extracted Cu 2p spectrum of the electrolyte.

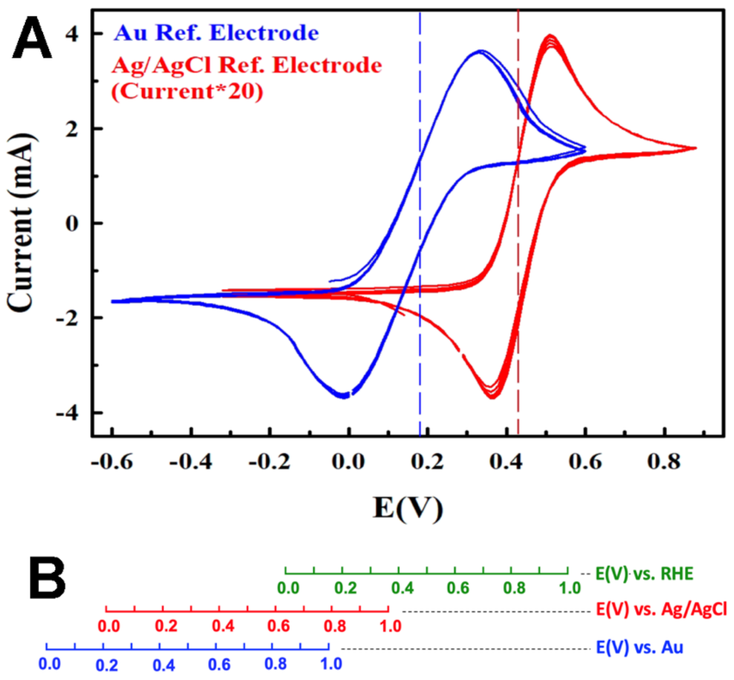

Supplementary Fig. 4. Voltage relationship between the in-cell Au pseudo-reference electrode and an Ag/AgCl electrode. (A) Cyclic Voltammograms (CVs) of 0.1M ferri/ferrocyanide solution using Au and Ag/AgCl as reference electrodes. (B) scale diagram of V_{Au} versus $V_{Ag/AgCl}$, V_{RHE} in in the ferri/ferrocyanide system (pH=7.3). Dashed lines are the equilibrium potential of the Au and Ag/AgCl electrodes.

Supplementary fig. 4A presents CV measurements of 0.1 M ferri/ferrocyanide solutions using Au and Ag/AgCl reference electrodes used to determine the relationship of the V_{Au} scale to that of the reversible hydrogen electrode (RHE). The CVs were measured in the potential window of +0.6 to −0.6 V_{Au} (*in-situ* cell) and +0.9 to −0.3 $V_{Ag/AgCl}$ (ex-situ experiment) with a scan rate of 20 mVs^{-1}. The potential difference between the Au pseudo-reference electrode and standard Ag/AgCl reference electrode is +0.21 V. This value is in good agreement with a previously reported results (+0.24 V) [1]. The measured pH of the 0.1M ferri/ferro cyanide solution was 7.3. The potential vs Ag/AgCl was converted to RHE and SHE using the Nernst equation [2], indicating a potential difference of 0.84 V vs RHE and 0.41 V vs SHE. Therefore, the potential difference between V_{Au} and standard reference electrodes ($V_{Ag/AgCl}$, V_{RHE}, V_{SHE}) in the ferri/ferrocyanide system with pH 7.3 (Supplementary Fig 3B) can be expressed as:

$$E_{Au} = E_{Ag/AgCl} - 0.21 = E_{RHE} - 0.84 = E_{SHE} - 0.41$$

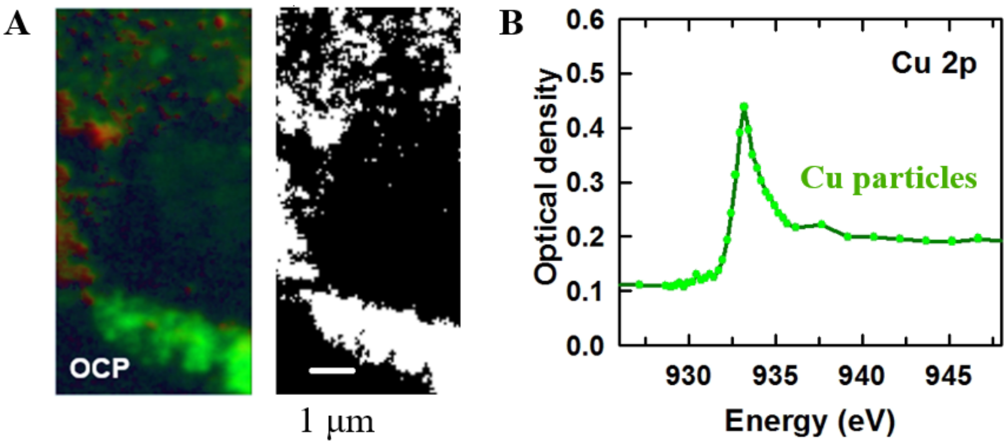

Supplementary Fig. 5. Chemical maps and Cu L_3 spectra of electro-deposited Cu particles. (A) (left) Map of Cu_2O derived by fitting a Cu L_3 stack to reference spectra of Cu, Cu_2O and CuO [3]. (right) Mask of the regions from which (B) the Cu L_3 spectrum was extracted. The solid dark green line is the spectrum of Cu_2O, while the light green points are the spectrum of the particles.

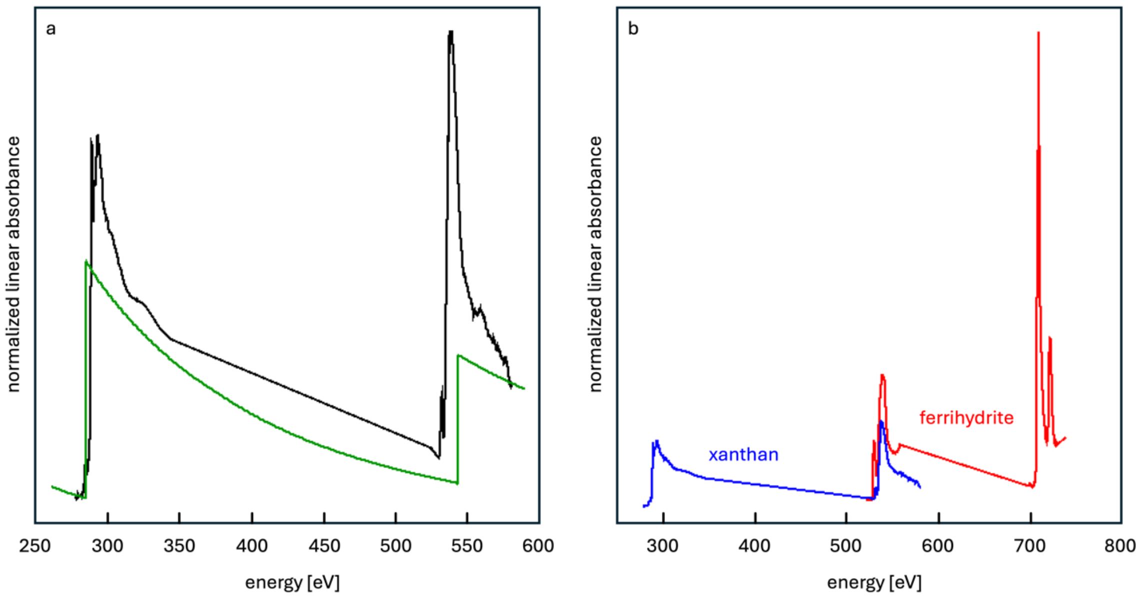

Supplementary Fig. 6. (a) Normalized NEXAFS spectrum of the reference polysaccharide xanthan across the C1s and O1s absorption edges (black line) and the respective edge-jump, calculated based on the atomic scattering factors for the elemental ratio CH_2O. (b) Normalized NEXAFS spectrum of the reference polysaccharide xanthan (blue) and ferrihydrite (red). The spectra were scaled 1.5 : 1.0 on thickness scale (assumed densities 1.0 for xanthan and 3.8 for ferrihydrite). The scaling was done to achieve similar edge jumps of the two compounds at the C1s and Fe2p absorption edges respectively, as observed in a previous study [4].

Supplementary Table 1. Energies of the main spectral features of ferri- and ferrocyanide at the Fe2p absorption edge.

A. $[Fe(CN)_6]^{3-}$(aq) Fe 3d occupancy in ground state: $(t_{2g})^5(e_g)^0$

	**Energy (eV)**				**Assignment [7] (d)**	
**Feature**	**This work**	**[5]**	**[6]**	**[7]**	**Fe L_3**	**Fe L_2**
1	706.2	706.2	706.2	705.6	$(t_{2g})^6(e_g)^0$	
2	710.2	710.3	710.4	710.0	$(t_{2g})^5(e_g)^1$	
3	712.5	712.6	712.7	712.5	$(t_{2g})^4(e_g)^2$	
4	718.9	719.2	719.1	718.9		$(t_{2g})^6(e_g)^0$
5	723.0	723.1	723.2	722.9		$(t_{2g})^5(e_g)^1$
6	726.1	726.2	726.2	725.9		$(t_{2g})^4(e_g)^2$

B. $[Fe(CN)_6]^{4-}$(aq) Fe 3d occupancy in ground state: $(t_{2g})^6(e_g)^0$

	**Energy (eV)**				**Assignment [7] (d)**	
**Feature**	**This work**	**[5]**	**[6]**	**[7]**	**Fe L_3**	**Fe L_2**
1	709.7	709.7	709.7	709.1	$(t_{2g})^6(e_g)^1$	
2	711.4	711.2	711.5	711.2	$(t_{2g})^5(e_g)^2$	
3	722.1	722.1	722.2	722.0		$(t_{2g})^6(e_g)^1$
4	724.1	724.0	724.2	723.9		$(t_{2g})^5(e_g)^2$

(d) dominant Fe 3d configuration in core excited state – see references cited in the text for details.

Supplementary Table 2. Source of the O1s NEXAFS spectra of pure reference compounds

**Compound**	**Source**	**Beamline**	**Reference**
Quinhydrone	Sigma-Aldrich	CLS,<br>10-ID1	this work
Protein (albumin)			[8]
Polysaccharide (Xanthan)			[9]
Suwannee River Humic Acid III	International Humic Substances Society	MaxIV Laboratories, SoftiMAX	this work
Ferrihydrite			[10]

Supplementary Information References

1 Ferrario, A., Scaramuzza, M., Pasqualotto, E., De Toni, A., Paccagnella, A. Development of a disposable gold electrodes-based sensor for electrochemical measurements of cDNA hybridization. *Procedia Chemistry* **6,** 36-45 (2012)

2 Niu, S., Li, S., Du, Y., Han, X., & Xu, P. How to reliably report the overpotential of an electrocatalyst. *ACS Energy Letters* **5**(4), 1083-1087, (2020)

3 Zhang, C., Eraky, H., Tan, S., Hitchcock, A., Higgins, D. In Situ Studies of Copper-Based CO2 Reduction Electrocatalysts by Scanning Transmission Soft X-ray Microscopy. *ACS Nano* **17,** 21337-21348, doi:10.1021/acsnano.3c05964 (2023)

4 Schmid, G., et al. Submicron-Scale Heterogeneities in Nickel Sorption of Various Cell−Mineral Aggregates Formed by Fe(II)-Oxidizing Bacteria. *Environ. Sci. Technol.* **50,** 1, 114–125, doi.org/10.1021/acs.est.5b02955, (2016)

5 Kitajima, Y., et al. Observation of π backbonding features appearing in Fe 2p X-ray absorption spectra and Fe 1s-4p-1s resonant X-ray emission spectra of RbMn [Fe (CN) 6]. In *Journal of Physics: Conference Series*, 012082. IOP Publishing (2013)

6 Risch, M., et al. Reversibility of Ferri-/Ferrocyanide Redox during Operando Soft X-ray Spectroscopy, *The Journal of Physical Chemistry C* **119**: 18903-18910 (2015)

7 Kunnus, K., et al. Viewing the valence electronic structure of ferric and ferrous hexacyanide in solution from the Fe and cyanide perspectives, *The Journal of Physical Chemistry B* **120**, 7182-7194 (2016)

8 Stewart-Ornstein, J., et al. Using Intrinsic X-ray Absorption Spectral Differences To Identify and Map Peptides and Proteins. *Phys. Chem. B* **111**, 26, 7691–7699. doi.org/10.1021/jp0720993 (2007)

9 Dynes, J.J., et al. Speciation and Quantitative Mapping of Metal Species in Microbial Biofilms Using Scanning Transmission X-ray Microscopy. *Environ. Sci. Technol.* **40**, 5, 1556–1565. doi.org/10.1021/es0513638 (2006)

10 Schmid, G., et al. 3-D analysis of bacterial cell-(iron)mineral aggregates formed during Fe(II) oxidation by the nitrate-reducing Acidovorax sp. strain BoFeN1 using complementary microscopy tomography approaches. *Geobiol.* **12**, 4, 340-361 doi.org/10.1111/gbi.12088 (2014)

Supplementary Movie 1: S1_platform_assembly.mp4
